# Biofilm Formation of *Staphylococcus aureus* from Pets, Livestock, and Wild Animals: Relationship with Clonal Lineages and Antimicrobial Resistance

**DOI:** 10.3390/antibiotics11060772

**Published:** 2022-06-04

**Authors:** Vanessa Silva, Elisete Correia, José Eduardo Pereira, Camino González-Machado, Rosa Capita, Carlos Alonso-Calleja, Gilberto Igrejas, Patrícia Poeta

**Affiliations:** 1 Microbiology and Antibiotic Resistance Team (MicroART), Department of Veterinary Sciences, University of Trás-os-Montes and Alto Douro (UTAD), 5000-801 Vila Real, Portugal; vanessasilva@utad.pt; 2 Department of Genetics and Biotechnology, University of Trás-os-Montes and Alto Douro (UTAD), 5000-801 Vila Real, Portugal; gigrejas@utad.pt; 3 Functional Genomics and Proteomics Unit, University of Trás-os-Montes and Alto Douro (UTAD), 5000-801 Vila Real, Portugal; 4 LAQV-REQUIMTE, Department of Chemistry, NOVA School of Science and Technology, Universidade Nova de Lisboa, 2829-516 Caparica, Portugal; 5 Center for Computational and Stochastic Mathematics (CEMAT), Department of Mathematics, University of Trás-os-Montes and Alto Douro (UTAD), 5001-801 Vila Real, Portugal; ecorreia@utad.pt; 6 CECAV—Veterinary and Animal Research Centre, University of Trás-os-Montes and Alto Douro (UTAD), 5000-801 Vila Real, Portugal; jeduardo@utad.pt; 7 Associate Laboratory for Animal and Veterinary Science (AL4AnimalS), University of Trás-os-Montes and Alto Douro (UTAD), 5000-801 Vila Real, Portugal; 8 Department of Food Hygiene and Technology, Veterinary Faculty, University of León, E-24071 León, Spain; mgonm@unileon.es (C.G.-M.); rosa.capita@unileon.es (R.C.); carlos.alonso.calleja@unileon.es (C.A.-C.); 9 Institute of Food Science and Technology, University of León, E-24071 León, Spain

**Keywords:** *S. aureus*, MRSA, animals, biofilms, antimicrobial resistance, genetic linages

## Abstract

This study aimed to compare the biofilm formation ability of *Staphylococcus aureus* isolated from a wide range of animals and study the association between biofilm formation and antimicrobial resistance and genetic lineages. A total of 214 *S. aureus* strains isolated from pets, livestock, and wild animals were evaluated regarding their ability to form biofilms by the microtiter biofilm assay and their structure via confocal scanning laser microscopy. Statistical analysis was used to find an association between biofilm formation and antimicrobial resistance, multidrug resistance, sequence types (STs), *spa* and *agr*-types of the isolates. The antimicrobial susceptibility of 24 h-old biofilms was assessed against minimum inhibitory concentrations (MIC) and 10× MIC of amikacin and tetracycline, and the biomass reduction was measured. The metabolic activity of biofilms after antimicrobial treatment was evaluated by the XTT assay. All isolates were had the ability to form biofilms. Yet, significant differences in biofilm biomass production were detected among animal species. Multidrug resistance had a positive association with biofilm formation as well as methicillin-resistance. Significant differences were also detected among the clonal lineages of the isolates. Both tetracycline and amikacin were able to significantly reduce the biofilm mass. However, none of the antimicrobials were able to eradicate the biofilm at the maximum concentration used. Our results provide important information on the biofilm-forming capacity of animal-adapted *S. aureus* isolates, which may have potential implications for the development of new biofilm-targeted therapeutics.

## 1. Introduction

*Staphylococcus aureus* is a ubiquitous organism that has the ability to colonize and infect humans and a wide range of other mammals and birds, with each host representing a distinct ecological niche [1]. *S. aureus* can cause nosocomial and community infections in humans which can range from skin and soft tissue infections to life-threatening infections such as endocarditis and bacteremia [2]. Over the past few years, antimicrobials have become less effective in treating *S. aureus* infections due to the frequently occurring antimicorbial resistance in these organisms, among which methicillin-resistant *S. aureus* (MRSA) are the most worrisome since MRSA strains are often resistant to several classes of antimicrobials [3]. While approximately 30% of the human population are colonized by *S. aureus*, in animals, this frequency varies with the host species [4]. It has been shown that the prevalence of *S. aureus* in chickens, pigs and sheep can be up to 90%, 42% and 29%, respectively, and in cows it ranges from 14 and 35% [5]. *S. aureus* has been detected in a taxonomically diverse range of animals, such as, carnivores, ruminants, lagomorphs, insectivore, reptiles and birds, living in a wide range of ecological niches [6,7,8,9,10,11,12,13,14]. Nevertheless, as in humans, *S. aureus* can also cause various types of infections in animals, the most common of which are mastitis in bovine, skin and soft tissue infections in companion animals, septic arthritis in poultry, abscesses in rabbits among others [15,16,17,18]. *S. aureus* from animals are also known to carry several antimicrobial resistance determinants that can be transferred to humans and other animals [1]. *S. aureus* can be grouped into many different genetic lineages defined by molecular typing methods, and it has been shown that some *S. aureus* lineages tend to be more prevalent in specific types of infections or be host-specific [19,20]. In fact, some studies shown that the healthcare-associated infections caused by livestock-associated MRSA clonal complex (CC) 398 were the result of a spillover from nearby pig farms [21,22]. *S. aureus* strains may also be transmitted to humans through the food chain via the fecal-oral route [23]. Studies have shown a high prevalence of staphylococci in food, such as, cheese and meat [24,25].

In addition to the antimicrobial resistance commonly found among *S. aureus* isolates, their ability to form biofilms plays an important role in the maintenance of the infection [26]. Biofilms provide protection to bacteria against environmental stresses, antimicrobials, disinfectants and host immune defenses [27]. Furthermore, mechanisms of antimicrobial resistance in biofilm cells are altered since bacteria susceptible to a given antimicrobial may increase the tolerance to an antimicrobial when grown in biofilms [28]. Biofilm formation is a dynamic and cyclic process which comprises three phenotypically distinct stages: attachment, maturation, and dispersion, although recently a few authors consider two more stages: multiplication and exodus [29,30]. Microbial surface components recognizing adhesive matrix molecules (MSCRAMMs) are the main family of proteins involved in *S*. *aureus* attachment [31]. The MSCRAMMs family includes many proteins such as fibronectin-binding protein A and B, clumping factors A and B, collagen-binding protein, laminin-binding protein, elastin-binding protein, and fibrinogen-binding protein, among others [27,32]. After the attachment, *S. aureus* maturate and accumulate in multilayered clusters surrounded by an extracellular matrix (ECM) which is the first line of defense and impairs the diffusion of antimicrobials [33]. Following biofilm maturation, cells within the biofilm can reactivate to a planktonic state through dispersal which can lead to new and subsequent infections in the host since bacterial cells can disseminate into the host and recolonize other available host sites [27,34]. In fact, biofilm are responsible for up to 80% of all human chronic and recurrent infections [35,36]. However, in veterinary medicine, only a few studies report the role of biofilm in animal infections [37,38,39]. In one of the most studied animal infections, mastitis, it has already been demonstrated that chronic and recurrent bovine mastitis share similar characteristics with chronic human infections [37]. Therefore, this study aimed to investigate the biofilm forming capacity of *S. aureus* and MRSA strains isolated from various animals, including pets, livestock, and wild animals. Furthermore, this study also aimed to investigate the biofilm-related genes in all strains and to find a possible correlation between the clonal lineage, phenotypic antimicrobial resistance, and host with the biofilm formation capacity of each isolate.

## 2. Materials and Methods

### 2.1. Study Design and Bacterial Isolates

Part of this work was a retrospective study that included 214 *S. aureus* strains, comprising *mec*A-MRSA, *mec*C-MRSA, and methicillin-susceptible *S. aureus* (MSSA) isolates. The isolates were recovered from animals’ infections and from healthy animals between 2018 and 2021: purulent lesions of pigs (*n* = 43), purulent lesions of farm rabbits (*n* = 16), healthy cattle (*n* = 36), quails (*n* = 29), hunting dogs (*n* = 11), camels (*n* = 11), Miranda donkeys (*n* = 4), wild hares (*n* = 3), wild rodents (*n* = 38) and wild owls (*n* = 23) [6,7,8,12,40,41,42]. All isolates were previously characterized regarding their antimicrobial resistance and genetic lineages by MLST, *spa*-and *agr*-typing which will be used to performed statistical analysis to study the correlations between biofilm formation (evaluated in this study) and antimicrobial resistance and genetic lineages of the isolates [6,7,8,12,40,41,42]. *S. aureus* ATCC^®^ 25923 (clinical isolate) was used as a positive control due to its great biofilm formation capacity. *S. aureus* ATCC^®^ 25923 is a lab strain that has been away from the physiological environment of the human host. The isolates were cryopreserved at −20 °C in skim milk.

### 2.2. Biofilm Formation Assay

The biofilm formation assay was performed as described elsewhere with some modifications [43]. Briefly, two colonies were transferred from fresh cultures to tubes containing 3 mL of Tryptic Soy Broth (TSB, Oxoid, Basingstoke, UK) and incubated at 37 °C for 16 ± 1 h with continuous shaking at 120 rpm (ES-80 Shaker-incubator, Grant Instruments, Cambridge, UK). After the incubation period, the bacterial suspension was adjusted to an optical density of 1 × 10^6^ colony forming units and 200 µL of bacterial suspension of different isolates were added to each well of the 96-well flat bottom microplate. *S. aureus* ATCC^®^ 25923 was included in all plates as a positive control. Fresh medium without bacterial inoculum was used as a negative control. The plates were incubated at 37 °C for 24 h without shaking. All experiments had seven technical replicates and were performed in triplicate.

#### 2.2.1. Biofilm Formation Assay

Biofilm mass was quantified using the Crystal Violet (CV) Staining method as previously described by Peeters et al. (2008), with some modifications [44]. After incubation, the plates were washed twice with 200 µL of distilled water to remove non-attached bacterial cells and plates were then allowed to dry at room temperature for approximately 2 h. Then, 100 µL of methanol (VWR International, Carnaxide, Portugal) was added to each well and incubated for 15 min for microbial biofilm fixation. Methanol was removed, the plates were allowed to dry at room temperature for 10 min. Then, 100 µL of CV at 1% (*v*/*v*) was added to each well for 10 min and after the CV solution was removed. The excess dye was removed by washing the plates twice with distilled water. Then, 100 µL of acetic acid 33% (*v*/*v*) was added to solubilize the CV and the absorbance was measured at 570 nm using a microplate reader BioTek ELx808U (BioTek, Winooski, VT, USA).

### 2.3. Confocal Laser Scanning Microscopy

Confocal laser scanning microscopy (CLSM) was used for the visualization of the biofilm aggregate structures. The isolates were selected according to their capacity of biofilm formation and antimicrobial resistance. A total of 12 strains representative of the bacterial collection were used: the most biofilm producer isolates of each group of animals and two *mec*C-MRSA strains.

The strains were grown in TSB for 24 h at 37 °C, and appropriate (two-fold) dilutions in the same culture broth were made to obtain a concentration of approximately 10^7^ cfu/mL. A volume of 250 μL of these cultures were added to the wells of Nunc™ MicroWell™ 96-Well Optical-Bottom Plates with Polymer Base (Thermo Fisher Scientific, Newington, NH, USA; reference number 165305). These are of high optical quality, and have a low fluorescent background and overall flatness, which allowing high resolution imaging. After one hour of adhesion at 37 °C, the wells were rinsed with 150 mM of NaCl in order to eliminate any non-adherent bacteria before being refilled with TSB. The plates were then incubated for 24 h at 37 °C. After incubation, the wells were rinsed with 150 mM of NaCl.

For staining with fluorescent dye, a volume of 1.00 µL of SYTO 9 (stock 20 mM in DMSO, Thermo Fisher Scientific, Madrid, Spain) was added to 1000 µL of NaCl 150 mM, and 250 µL of this solution was put into each well. The plate was then incubated in the dark at 37 °C for 20 min to enable fluorescent labelling of the bacteria.

Confocal laser scanning microscopy (CLSM) image acquisition was performed using a Zeiss LSM 800 Airyscan confocal laser scanning microscope with ZEN 2.3 software (Carl Zeiss, Jena, Germany). Channel mode visualization was done using the 63× (0.8 NA) objective with oil immersion. The microscopic parameters used for SYTO9 stained cells was previously reported [45]. Stacks of horizontal plane images (512 × 512 pixels corresponding to 126.8 μm × 126.8 μm) with a z-step of 0.25 μm, were acquired for each well from three different randomly chosen areas. Three independent experiments were performed for each strain on separate days. For image analysis, original Zeiss files (CZI format) were imported into the IMARIS 9.1 software package (Bitplane, Zurich, Switzerland) for modelling in three dimensions. Biovolume represented the amount of biofilm (μm^3^) in the observation field of 16,078.2 μm^2^. Surface coverage (%) reflected the efficiency of substratum colonization by the populations of bacteria. Roughness provided a measure of how much the thickness of the biofilm varied and was thus an indicator of biofilm heterogeneity. A roughness with a value of zero indicates a biofilm of uniform thickness, and the greater the roughness coefficient, the rougher the surface. The maximum thickness (μm) of biofilms was determined directly from the confocal stack images. 

### 2.4. Effect of Antimicrobials on 24 h-Old Biofilms

A total of 23 strains representative of the bacterial collection, the most and the least biofilm producer isolate of each group of animals and the three *mec*C-MRSA strains, were used to investigate the efficacy of conventional antimicrobials on the reduction of biofilm mass. The individual percentage of biofilm formation and phenotypic resistance of the selected isolates is shown in Table 1. The minimal inhibitory concentrations (MICs) of amikacin and tetracycline were determined by a standard broth microdilution method in sterile 96-well microplates according to the EUCAST guidelines and as described by Silva et al. [46,47]. Biofilm formation was carried out as described in Section 2.2. After the incubation period and the formation of the 24 h-old biofilms, the medium was replaced by 200 µL of TSB supplemented with amikacin or tetracycline (to a final concentration at MIC and 10 × MIC) and incubated at 37 °C for 24 h without shaking. After incubation with antimicrobial agents, biofilm mass was quantified using the CV staining method as described in Section 2.2.1. All experiments had four technical replicates.

#### Effect of Antimicrobials on Metabolic Activity

The effect of antimicrobials on metabolic activity of biofilms was determined by the XTT colorimetric method. Briefly, the reaction solution was prepared by adding 0.1 mL of PMS (*n*-methyl dibenzopyrazine methyl sulfate) to 5 mL of XTT reagent according the instrutions of the cell proliferation assay kit (XTT Kit, Appli Chem Panreac). After the incubation period with antimicrobials, biofilms were washed with 200 µL of 0.9% (*w/v*) NaCl solution and 50 μL of the reaction solution was added to each well. The plates were incubated for 5 h and the absorbance was measured with a microplate reader (BioTek ELx808U, Winooski, VT, USA) at a wavelength of 490 nm.

### 2.5. Statistical Analysis

Descriptive statistics of data are presented as mean (M) and standard deviation (SD) when appropriate. Skewness and kurtosis coefficients were computed for univariate normality analysis purposes. To determine whether the resistance, multi-resistance phenotypes of the isolates and the clonal lineages had a statistically significant effect on biofilm a one-way analysis of variance (ANOVA) were performed followed by Tukey’s pos-hoc tests, when appropriated. To determine whether biofilm formation is influenced by resistance to a particular antimicrobial, an independent *t*-test was used. All statistical analysis was performed using SPSS (IBM SPSS Statistics 26, SPSS Inc., Chicago, IL, USA). Statistically significant effects were assumed for *p* < 0.05.

## 3. Results

### 3.1. Biofilm Formation

**Figure 1:**
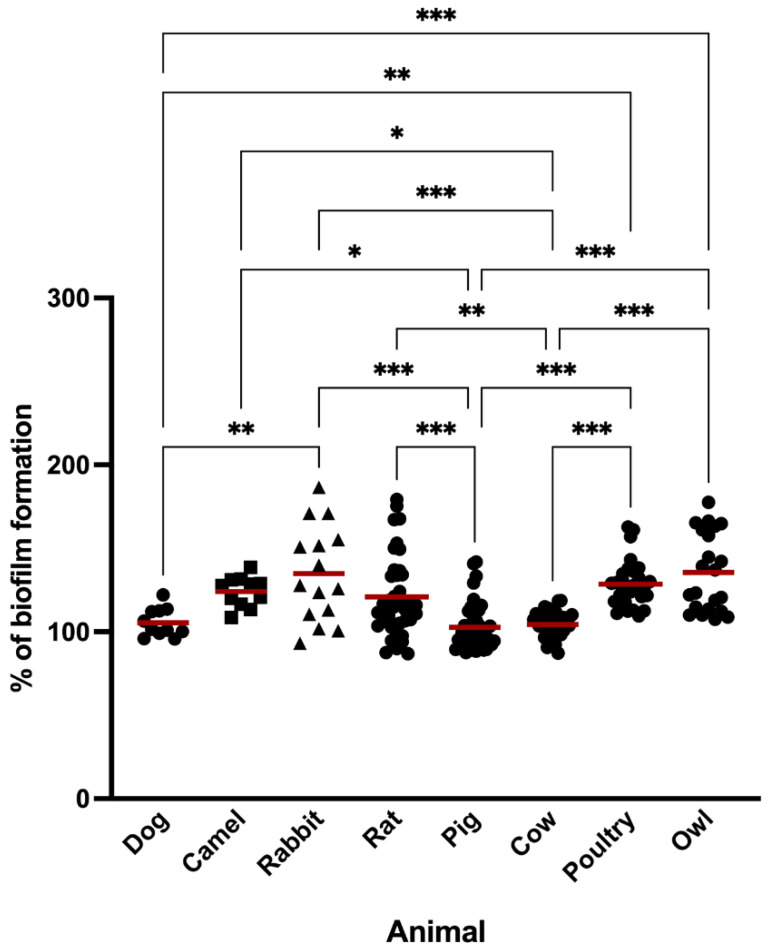
Biofilm formation capacity of *S. aureus* strains isolated from different animal species. The symbols represent the biomass mean of the biofilm formed in independent tests of the individual isolates. The red lines represent the average of biofilm mass formed by all isolates. Statistical significance was determined using Tukey’s multiple comparison test (* *p* < 0.05; ** *p* < 0.01; *** *p* < 0.001).

All strains isolated from different animal species produced biofilm. The results were normalized against *S. aureus* ATCC 25923 so that the comparison of results could be more consistent. Figure 1 shows the biofilm formation of each isolate grouped by animal host. *S. aureus* strains isolated from rabbit infections were the ones that produced the most biofilm with a percentage mean of biofilm formation of 138.69 ± 33.02 which was significantly higher than dog, pig, and cow isolates (*p* < 0.01 and *p* < 0.001). The strains that produced less biofilm mass were those isolated from pigs and cows which included both MRSA and MSSA strains. The percentage mean of pigs and cows’ isolates quite similar among these and significantly lower than most strains isolated from camel (*p* < 0.05), rabbit (*p* < 0.001), rodents (*p* < 0.001 and *p* < 0.01), poultry (*p* < 0.001) and wild owls (*p* < 0.001). The most and least biofilm-producing strains belong to the rabbits and cows’ groups and the mean percentage of biofilm production was 87.18 and 186.5, respectively. *S. aureus* isolated from donkeys and wild hares were not included in the statistical analysis since the number of isolates was not representative (*n* = 4 and *n* = 3, respectively). However, biofilm formation was also investigated in these isolates and the mean percentage was 130.10 ± 22.14 and 133.79 ± 17.09 for donkeys and hares’ isolates, respectively.

### 3.2. CLSM Analysis

**Figure 2:**
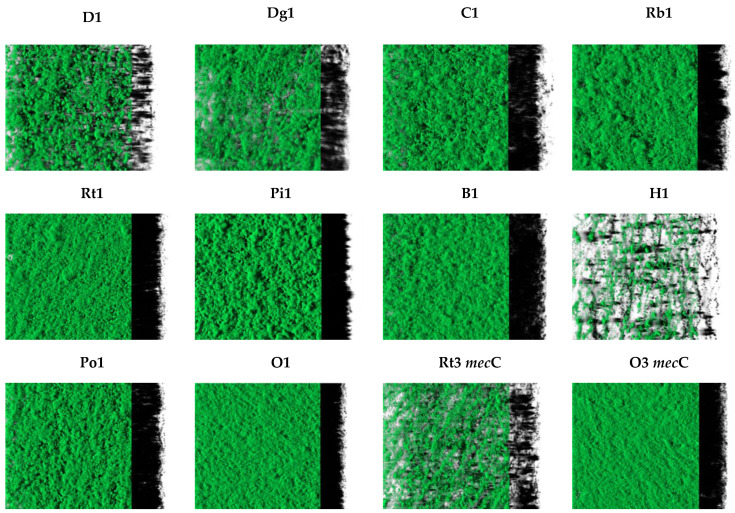
The images (126.8 µm × 126.8 µm) correspond to three-dimensional reconstructions obtained by CLSM and processed with the IMARIS 9.1 software, including the virtual projections of the shadows on the right. D: donkey; Dg: dog; C: camel; Rb: rabbit; Rt: rodent; Pi: pig; B: bovine; H: hare; Po: poultry; O owl.

The architecture of biofilms formed by 12 *S. aureus* isolates was evaluated after 24 h of incubation at 37 °C. Figure 2 shows the representative structures of the biofilms formed. Strong variations between isolates were detected regarding the three-dimensional structures of biofilms. *S. aureus* isolated from donkey (D1), dog (Dg1), wild hare (H1) and the *mec*C strain isolated from wild rodents (Rt3 *mec*C) formed biofilms containing several small aggregates. The remaining strains produced rough biofilms with dense and homogeneous structures that covered the entire available surface after 24 h of incubation.

**Figure 3:**
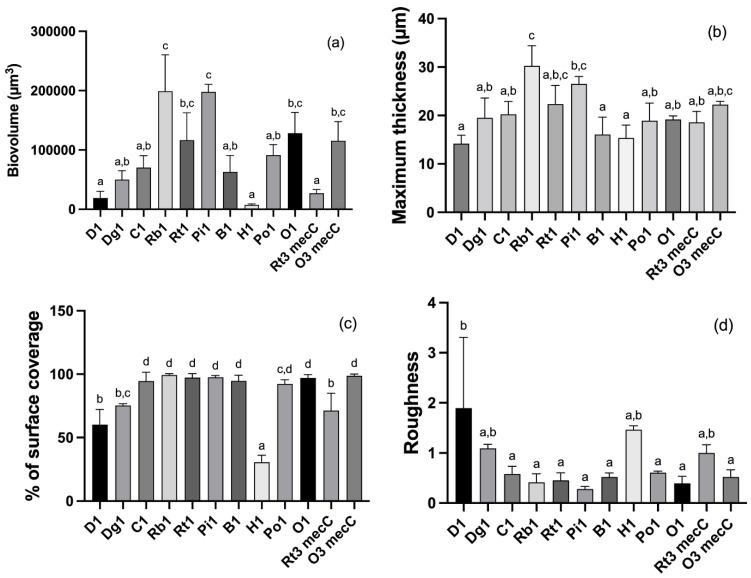
Biovolume in the observation field of 16,078.2 μm^2^ (**a**), maximum height (**b**), percentage of surface area covered (**c**) and roughness (**d**) of biofilms formed de 12 selected *S. aureus* isolates. Statistical significance was determined using Tukey’s multiple comparison test. The values marked with the same letter are not statistically significant as determined by the Tukey’s post hoc test *(p* < 0.05). D: donkey; Dg: dog; C: camel; Rb: rabbit; Rt: rodent; Pi: pig; B: bovine; H: hare; Po: poultry; O owl.

CLSM together with quantitative image analysis allows the determination of the structural parameters of the biofilms, allowing the quantitative comparison of biofilms of different strains. The biovolume, the percentage of covered surface, the maximum height and the roughness were determined, and the results are shown in Figure 3. Significant differences were obtained among the isolates, with a lower biovolume in strains D1, H1 and Rt3 *mec*C (mean of 18,783.97 ± 11,282.27, 7260.78 ± 1839.67 and 27,207.42 ± 6280.79 μm^3^, respectively, in the observation field of 16.078.2 μm^2^) compared to the biofilms formed by strains MRSA strains Rb1 (199,074.89 ± 61,222.63 μm^3^) and Pi1 (197,837.76 ± 12,770.93 μm^3^) (*p* < 0.001). Accordantly, the biofilm formed by Rb1 and Pi1 strains had significantly higher thickness (30.25 and 26.5 µm, respectively) than most strains from other origins. MRSA Rb1 (30.25 ± 4.18 µm) isolate formed biofilms with significant higher maximum height than other isolates, particularly than MRSA H1 (15.33 ± 2.67 µm) (*p* < 0.0001). Regarding the percentage of surface coverage, MRSA strain H1 showed significantly lower coverage than the other isolates (*p* < 0.001). The roughness was higher in D1 biofilm than in most of the other isolates (*p* < 0.05 and *p* < 0.01).

### 3.3. Antimicrobial Resistance and Biofilm Formation

**Figure 4:**
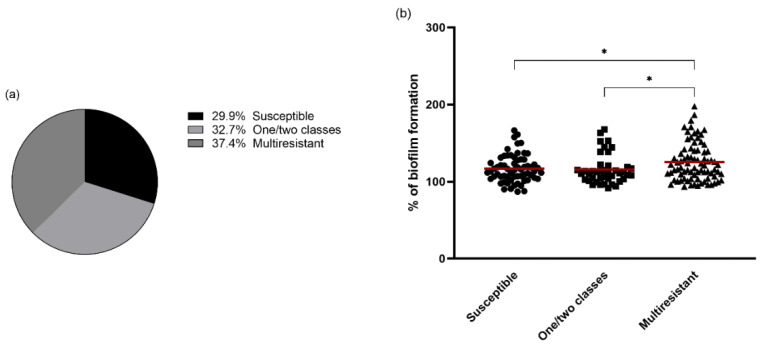
(**a**) Percentage isolates susceptible to all antimicrobials, resistant to one or two classes of antimicrobials and MDR. (**b**) Mean of biofilm formation among isolates susceptible to antimicrobials, resistant to one/two classes and MDR isolates. The red lines represent the average of biofilm mass formed by all isolates. Statistical significance was determined using Tukey’s multiple comparison test (* *p* < 0.05).

Relationship between the biofilm-forming capacity and antimicrobial resistance of *S. aureus* isolates was investigated by statistical analysis using Tukey’s multiple comparisons and T Student’s *t*-test. First, the composition of the biofilm formation groups with respect to resistance and multi-resistance phenotypes was investigated. Among all isolates studied, 64 (29.9%) were susceptible to all antimicrobial classes, 70 (32.7%) were susceptible to one or two classes and 80 (37.4%) were resistant to three or more classes (multi-drug resistant). As shown in Figure 4, multidrug-resistant (MDR) isolates produced more biofilm than isolates from the other two groups (*p* < 0.05). Susceptible isolates and isolates resistant to one or two classes of antimicrobials produced similar amounts of biofilm biomass. Analyzing the difference in mass between MDR and non MDR, MDR isolates produced more biofilm (*p* < 0.01).

To determine whether biofilm formation is related with resistance to any particular antimicrobial, the biofilm-forming capacities was evaluated in isolates with different resistance profiles to 11 antimicrobials. Isolates susceptible/resistant to penicillin (*n* = 92 and *n* = 122), cefoxitin (*n* = 120 and *n* = 94), ciprofloxacin (*n* = 127 and *n* = 87), gentamycin (*n* = 44 and *n* = 170), tobramycin (*n* = 38 and *n* = 176), kanamycin (*n* = 45 and *n* = 169), erythromycin (*n* = 144 and *n* = 70), clindamycin (*n* = 144 and *n* = 70), tetracycline (*n* = 143 and *n* = 71), chloramphenicol (*n* = 192 and *n* = 22)and fusidic acid (*n* = 199 and *n* = 15) were included. The results revealed that isolates resistant to penicillin, tobramycin, tetracycline and fusidic acid produced similar biofilm mass than isolates susceptible to those antimicrobials (Table 2). Nevertheless, a significant higher biofilm production was shown in isolates resistant to cefoxitin (MRSA), ciprofloxacin, gentamicin, kanamycin, erythromycin, and clindamycin when compared to isolates susceptible to those antimicrobials. In contrast, isolates susceptible to chloramphenicol produced stronger biofilms than resistant ones (*p* < 0.05).

### 3.4. Relation between Molecular Typing and Biofilm Formation

**Figure 5:**
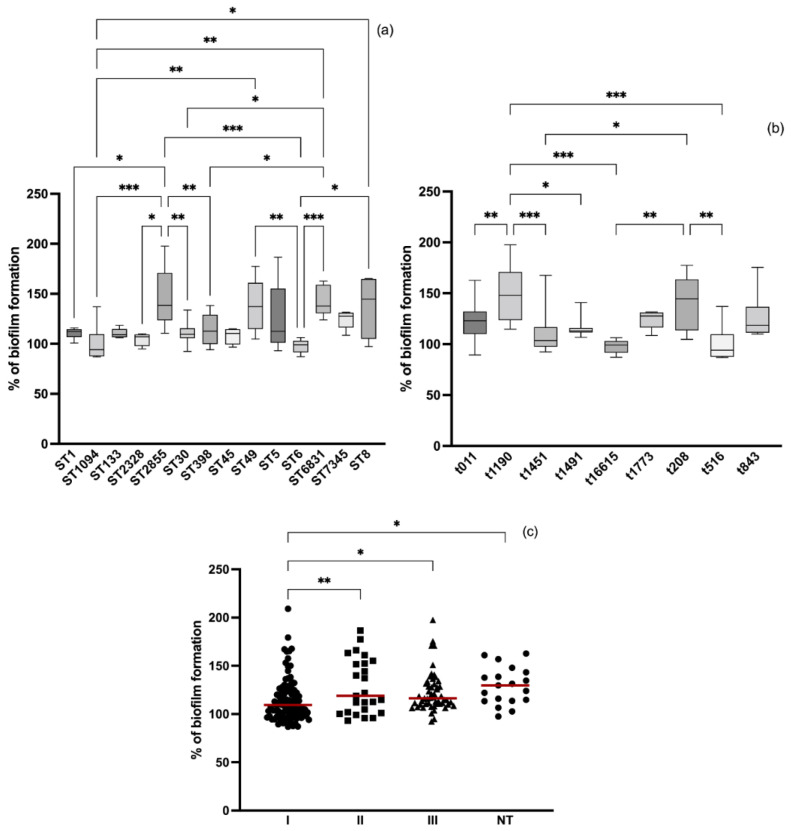
Biofilm formation among isolates grouped into different clonal lineages. Relationship between biofilm formation and: (**a**) STs; (**b**) *spa*-types and (**c**) *agr* types. The red lines represent the average of biofilm mass formed by all isolates. Statistical significance was determined using Tukey’s multiple comparison test (* *p* < 0.05; ** *p* < 0.01; *** *p* < 0.001).

To evaluate whether the clonal lineages of the isolates influence the capacity of biofilm formation, the isolates were divided into their sequence types (STs), *spa*- and *agr*-types. We considered that some isolates belonged to a wide range of clonal lineages, some of which had only a few associated isolates, and in order to have a balanced experimental design in which there is a similar number of isolates in all groups, isolates belonging to the most prevalent STs, *spa*- and *agr*-types were selected. The isolates used in this study were ascribed to 39 STs but only 14 STs were analyzed. The relationship between STs groups and biofilm formation is shown in Figure 5a). To standardize the results, biofilm formation of each isolate was normalized according to the results obtained from the positive control strain *S. aureus* ATCC^®^ 25923. Isolates belonging to ST2855 (*n* = 11), ST49 (*n* = 11), ST6831 (*n* = 9) and ST8 (*n* = 7) produced similar amounts of biomass among them and higher than most of the other STs isolates. Isolates belonging to ST1094 (*n* = 7) produced the least amount of biomass with a mean percentage of biofilm production of 99.09 ± 18.09. With regards to *spa*-type, 56 types were identified among the 214 isolates but only 9 types were selected as the most common (Figure 5b). Isolates ascribed to *spa*-type t1190 (*n* = 7) produced the most biofilm biomass (152.06 ± 11.11) and the amount of biomass was significantly higher than that of the isolates that belonging to t011 (*n* = 11) (*p* < 0.01), t1451 (*n* = 11) (*p* < 0.001), t1491 (*n* = 8) (*p* < 0.05), t16615 (*n* = 7) (*p* < 0.01) and t516 (*n* = 7) (*p* < 0.01). Finally, *S. aureus* isolates used in this study were ascribed to the four *agr* types and some were not typeable: However, the three isolates belonging to *agr* IV were not included in the statistical analysis. Isolates belonging to *agr* type I (*n* = 33) produced significantly lower amount of biomass than isolates belonging to ***agr*** II (*n* = 42), *agr* III (*n* = 25) and non-typable (*n* = 19) isolates (Figure 5c).

### 3.5. Effect of Antimicrobials on 24 h-Old Biofilms

**Figure 6:**
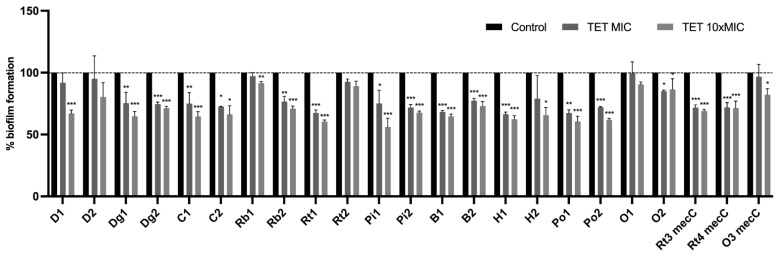
Effect of tetracycline on the biofilm biomass reduction of 23 isolates at MIC and 10× MIC. Data are presented as mean ± standard deviation for four independent replicates. Statistical significance was determined using Dunnett’s multiple comparison test (* *p* < 0.05; ** *p* < 0.01; ***p < 0.001). D: donkey; Dg: dog; C: camel; Rb: rabbit; Rt: rodent; Pi: pig; B: bovine; H: hare; Po: poultry; O owl.

**Figure 7:**
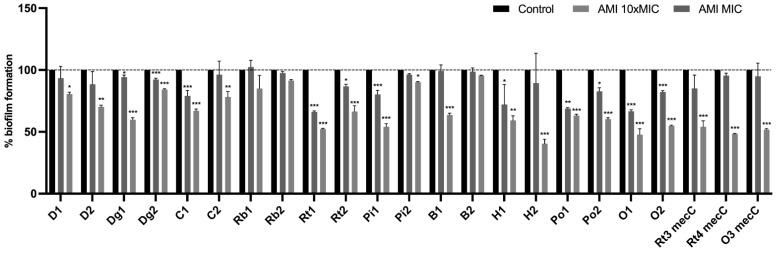
Effect of amikacin on the biofilm biomass reduction of 23 isolates at MIC and 10× MIC. Data are presented as mean ± standard deviation for four independent replicates. Statistical significance was determined using Dunnett’s multiple comparison test (* *p* < 0.05; ** *p* < 0.01; *** *p* < 0.001). D: donkey; Dg: dog; C: camel; Rb: rabbit; Rt: rodent; Pi: pig; B: bovine; H: hare; Po: poultry; O owl.

To assess whether biofilm-specific resistance influences the action of conventional antimicrobials, the MICs of tetracycline and amikacin were determined in the same set of 23 isolates used in CLSM analysis. The MICs for these isolates ranged from 0.052 to 64 μg/mL for tetracycline and from 0.5 to 64 μg/mL for amikacin. Then, the capacity of these antimicrobials, at concentrations of MIC and ten times MIC (10× MIC), to reduce pre-established 24-h-old biofilms was evaluated using the microtiter biofilm assay. Results were normalized according to the 48-h-old biofilm mass recorded for each strain tested grown without the presence of antimicrobials. As shown in Figure 6, all isolates treated with 10× MIC had a significant decrease in biofilm mass except for strain D2 which is a donkey isolate with phenotypic resistance to tetracycline. In fact, 15 out of 23 isolates had a very highly significant biomass reduction when treated with 10× MIC (*p* < 0.001). The biofilm biomass of the isolates was also reduced using the MIC concentration, with the exception of isolate O1, with a very highly significantly reduction in 8 isolates. The strains isolated from wild owls, O1 and O2, had an unusual result since in biofilm mass of isolate O1 suffered a slight increase when treated with MIC concentration and the biofilm mass treated with 10× MIC of isolate O2 was slightly higher than the biofilm treated with MIC concentration. Nevertheless, those differences were not statistically significant. Results for the 24 h-old biofilm treatment with amikacin are shown in Figure 7. Amikacin at 10× MIC was able to reduce the biofilm mass in all isolates with very highly significant reduction in 15 isolates (*p* < 0.001). Amikacin at MIC concentration was also able to significantly reduce the biomass of 12 isolates. Biomass reduction was not identified in 3 isolates and in isolate Rb1 there was even an increase in biomass after treatment at MIC. Overall, amikacin at 10× MIC was the antimicrobial that had the greatest influence on the reduction of biomass being higher than tetracycline at 10× MIC (*p* < 0.05). Highly significant differences in biofilm reduction were observed between amikacin at MIC and 10× MIC and tetracycline at MIC and 10× MIC (*p* < 0.001). There were no significant differences between both antimicrobials in biomass reduction at MIC concentration.

#### Effect of Antimicrobials on Metabolic Activity

**Figure 8:**
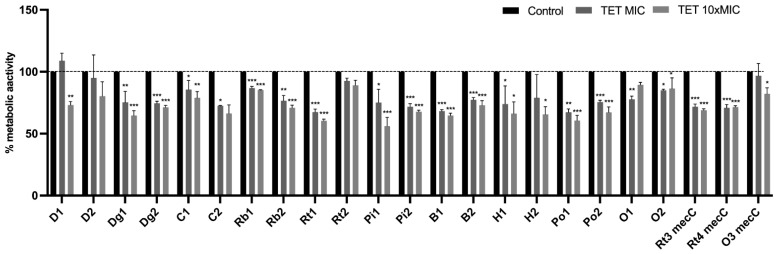
Metabolic activity of *S. aureus* biofilms before and after treated with tetracycline at concentrations of MIC and 10× MIC. The results are expressed as percentage of metabolic activity. Statistical significance was determined using Dunnett’s multiple comparison test (* *p* < 0.05; ** *p* < 0.01; *** *p* < 0.001). D: donkey; Dg: dog; C: camel; Rb: rabbit; Rt: rodent; Pi: pig; B: bovine; H: hare; Po: poultry; O owl.

**Figure 9:**
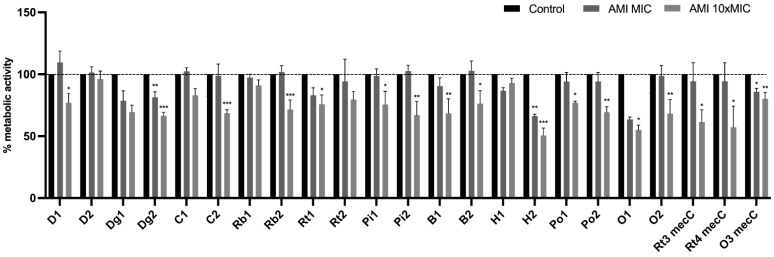
Metabolic activity of *S. aureus* biofilms before and after treated with amikacin at concentrations of MIC and 10× MIC. The results are expressed as percentage of metabolic activity. Statistical significance was determined using Dunnett’s multiple comparison test (* *p* < 0.05; ** *p* < 0.01; *** *p* < 0.001). D: donkey; Dg: dog; C: camel; Rb: rabbit; Rt: rodent; Pi: pig; B: bovine; H: hare; Po: poultry; O owl.

The XTT assay was used to evaluate the biofilms’ metabolic activity after treatment with tetracycline and amikacin at concentrations of MIC and 10× MIC. The results were normalized according to the 48 h-old biofilm of each tested isolate which were grown without the presence of antimicrobials. The effect of tetracycline on the metabolic activity of biofilms are shown in Figure 8. There was a significant decrease in biofilms metabolic activity of 18 isolates treated with tetracycline at 10× MIC which is in accordance with the results obtained in the biofilm mass reduction. The only two isolates that did not suffered a significant reduction in the metabolic activity with both concentrations were D2 and Rt2 which also did not suffer a biomass reduction with any of the concentrations. In fact, there was an increase, although not significant, in the metabolic activity of the D2. As with the biomass reduction assay, the O1 and O2 isolates demonstrate atypical behavior with an increase in metabolic activity at a concentration of 10× MIC in relation to the MIC. Regarding the effect of amikacin on the metabolic activity of biofilms, there was a significant reduction in 17 isolates at 10× MIC and in 5 isolates at MIC concentration (Figure 9). Increase in the metabolic activity after amikacin treatment was observed only at MIC concentration in isolates D1, D2, C1, Rb2, Pi2 and B2. However, no increase was statistically significant.

## 4. Discussion

*S. aureus* is recognized as one of the most frequent causes of biofilm-associated infections. It is suggested to be responsible for about 80% of all chronic human infections [48]. However, the role of biofilm formation in animal infections is not well understood, since biofilm studies in animals are uncommonly reported, and most studies conducted so far focus mainly on bovine mastitis [37,49]. Animal-adapted *S. aureus* has different characteristics and may behave differently from human *S. aureus*, for example, *S. aureus* ST398 in animals are usually associated with methicillin- and tetracycline-resistance [50]. Therefore, the impact of biofilm formation of animal-associated *S. aureus* strains cannot be ignored.

In this study, biofilm formation of 214 *S. aureus* isolated from several animal species was evaluated. As far as we know, this is the first study comparing the biofilm-forming capacity of *S. aureus* between a wide range of animals, including pets, livestock and wild animals, since most studies focus only in one animal. In this study, all *S. aureus* isolates were biofilm producers and the biofilm formation differed greatly among animals (Figure 1). Indeed, microscopic observations revealed marked variability in biofilm formation and structure from different strains (Figure 2). Studies have shown that food-producing animals are often colonized by strong biofilm-producer strains [51,52]. In our study, isolates from food-producing rabbits and poultry were strong biofilm-producers which may impose a public health concern since biofilm formation represents a major risk in the food sector as well as high economic losses in the livestock industry [53]. A study conducted in Brazil showed that the majority of *S. aureus* isolated from raw poultry meat were biofilm-producers [54]. Another study with poultry meat reported the presence of extremely strong biofilm-producing strains isolated from chicken meat products [55]. Nevertheless, in our study, *S. aureus* isolated from pigs and cows were the least biofilm-producers among all tested animals. Concordant results were obtained by Rodríguez-López et al., who studied the presence of biofilm-forming MRSA in pigs and showed that, although most isolates were biofilm-producers, most of the producers displayed low levels of biofilm production [52]. Studies conducted with *S. aureus* isolated from dairy also showed that most isolates were weak-biofilm producers [56,57,58]. In our study, *S. aureus* isolates colonizing wild animals were strong biofilm producers. However, only a very limited number of studies reported the biofilm formation capacity of free-living wild animals’ strains. In a study conducted with wild animals undergoing rehabilitation, 72.5% of the *S. aureus* isolated were capable of forming biofilms [59].

In this study, the relationship between biofilm formation and antimicrobial resistance in all isolates was also evaluated. Isolates with MDR phenotypes were significantly stronger biofilm-producers than isolates with no resistance or showing resistance to one or two antimicrobial classes (*p* < 0.01). Previous studies regarding the relationship between biofilm formation and antimicrobial resistance have yielded different results among them. In accordance with our results, a study conducted with *S. aureus* from beef meat detected a positive relationship between antimicrobial resistance and strong biofilm-producing strains [60]. Lin et al., also evaluated the biofilm formation according to susceptibility, resistance to one and two antimicrobial classes and MDR isolates and reported similar reports to ours [61]. Neopane et al., and Ou et al., also concluded that the biofilm-forming strains had a higher tendency to exhibit MDR [62,63]. However, other authors claim that there is no significant difference in the formation between MDR and non-MDR strains [52,64]. Therefore, the influence of antimicrobial resistance on biofilm formation may be due to resistance to a particular set of antimicrobials. In our study, isolates resistant to cefoxitin, gentamycin, kanamycin, tetracycline, erythromycin, and clindamycin formed significantly higher amounts of biofilm biomass than susceptible isolates. Furthermore, the major difference was detected between ciprofloxacin resistant and susceptible isolates (*p* < 0.001). Neopane et al., also reported that resistant rates of biofilm producers to ciprofloxacin were significantly higher than those of biofilm nonproducers (*p* < 0.05) [62]. These results may indicate importance of antimicrobial resistance to individual antimicrobials in the pathogenesis of biofilm-producing strains. Furthermore, a significant relationship between methicillin resistance and biofilm formation was found as previously reported [65]. On the other hand, isolates resistant to chloramphenicol produced weaker biofilm than isolates showing susceptibility (*p* < 0.05). Gaire et al., reported that most isolates susceptible to ciprofloxacin, gentamycin, clindamycin and cefoxitin were weak biofilm producers. In contrast, in the same study, it was reported that most isolates resistant to penicillin and erythromycin produced weak biofilms [66]. In contrast to our results, Sun et al., showed that isolates resistant to tetracycline were stronger biofilm producers than susceptible isolates [67]. These differences may be due to other strain characteristics such as the clonal lineages.

Most of our tetracycline-resistant isolates belonged to ST398 which was related with isolates producing smaller amounts of biofilm mass when compared to other STs, despite the fact that most isolates were multidrug resistant (Figure 5). In the study of Chen et al., *S. aureus* belonging to ST398 showing resistance to multiple antimicrobials were also weak biofilm-producers [68]. Isolates ascribed to ST2855 produced significantly more biomass than most of the other STs and were mostly MDR. ST2855 is animal associated and has been frequently reported in lagomorphs but none of the studies investigated the ability to form biofilms [13,69]. Other isolates belonging to animal associated STs included in this study, such as, ST49 and ST6831, were also strong biofilm producers. However, information on the production of biofilm from strains of animal origin is still very scarce. The isolates belonging to ST8 were also strong biofilm producers. ST8 is an human associated ST and is frequently related with methicillin-resistance in community humans and animals [7]. Other studies have shown that ST8 associated with food and human infections generally produces strong biofilms [70,71]. In fact, it has been suggested that isolates belonging to CC8 have a predisposition to produce strong biofilms and increased adherence [72,73]. However, in this study, other human related STs, such as ST6, ST45 and ST30, produced significantly lower amounts of biofilm biomass. Accordantly, Naicker et al., reported that *S. aureus* isolates belonging to CC30 and ST45 were weak to non-producers of biofilm [70]. However, ST6 was reported as a strong biofilm producer in the same study. Vitale et al., reported that isolates derived from animal samples were weak or moderate biofilm producers while isolates from humans were strong producers [74]. *spa*-types t1190 and t208 isolates significantly produced more biofilm mass than most isolates of the other *spa*-types. This result was excepted since t1190 and t208 are associated with ST2855 and ST49, respectively. Opposite results were observed in two studies that reported t1190 isolates from bovine mastitis as weak to moderate biofilm producers [75,76]. Regarding the *agr* types, isolates belonging to type I significantly produced lower amounts of biofilm biomass than types II and III and non-typable isolates. The association between biofilm formation and the *agr* types varied between studies and some studies did not even find any association [51,77,78,79]. It has been noted, however, that *S. aureus* strains negative for *agr* are able to initiate and establish colonization since inhibiting the *agr* system promotes biofilm formation [80,81]. In this study, although not statistically significant, *agr*-negative isolates had a biofilm formation median higher than *agr*-positive isolates.

*S. aureus* biofilm infections are recalcitrant to clearance by antimicrobials [82]. The mechanisms underlying biofilm resistance are diverse, including lack of antimicrobial diffusion into the biofilm matrix, expression of efflux pump and persister cells [51,83]. In fact, biofilm cells can be 10 to 1000 times less susceptibility to antimicrobials than their planktonic counterparts [84]. In this study, the effect of two antimicrobials used in medicine veterinary, tetracycline and amikacin, on the reduction of biofilm biomass formed by *S. aureus* isolated from animals was tested (Figure 6 and Figure 7). The concentrations of the antimicrobials were assessed for each isolate according to their MIC. As expected, none of the antimicrobials was able to eradicate the biofilm completely, not even at concentration of 10× MIC. Nevertheless, the biofilms of some isolates suffered reductions above 50% in biomass after exposure 10× MIC of amikacin (isolates H2, O1 and Rt4*mec*C). The mode of action of amikacin, an aminoglycoside, is diffusing through the outer membrane of the bacteria and binding the 30S ribosomal subunit interfering with bacterial growth [85]. In the study of Baishya et al., the MIC and minimum biofilm inhibitory concentration was 0.25 and 8 μg/mL, respectively, which shows that concentration superior to 10× MIC was required to eradicate *S. aureus* biofilms using amikacin as also showed in our study [86]. Biofilm matrix restricts the of antimicrobials diffusion [27]. However, Singh et al., demonstrated that he penetration of amikacin remains unaffected [87]. In another study, amikacin was used against *S. aureus* biofilms and the authors concluded that the increased resistance of biofilms is largely dependent on the presence of persister cells [88]. Indeed, biofilms contain more persister cells which have lower growth rates and, therefore, are less susceptible by antimicrobials [89]. Tetracycline-treated biofilms did not undergo such an abrupt reduction as with amikacin, but overall, this antimicrobial caused significant reductions on biofilm mass at 10× MIC. It has been demonstrated that the antimicrobial class of tetracyclines has efficacy against *S. aureus* biofilms [90]. Indeed, studies have shown that antimicrobials that target protein or RNA synthesis, such as tetracycline, have higher efficiency than antimicrobials with other types of modes of action such as cell wall synthesis [91,92]. Furthermore, tetracycline suppresses the localization of the autolysin Atl which is associated with the biofilm initial attachment [93,94].

Although the CV assay is a reliable method to quantify the biofilm biomass, it quantifies the matrix of both living and dead cells [95]. Therefore, the XTT assay was used to determine the metabolic activity of biofilm cells after treatment with antimicrobials. Most biofilms that underwent a reduction in biomass after antimicrobial treatment also demonstrated a lower metabolic activity. However, it has been shown that, although the XTT assay is a reliable and rapid method, it may have lower sensitivity than other methods, such as, colony forming units plating method in biofilm assays [96,97]. Indeed, in our study, some strains, particularly when treated with tetracycline MIC, increased their metabolic activity. However, it is not possible to state whether this increase is due to the higher number of biofilm living cells, if the biofilm cells increased their metabolism in an attempt to resist the external pressure caused by antimicrobials or if the cells might have been at the proliferative stage, with a reduced extracellular matrix [32].

## 5. Conclusions

In this study, all *S. aureus* isolated from a wide range of animals had the ability to form biofilms. However, significant differences were observed between biofilms produced by *S. aureus* from different animal species. An association between biofilm formation and antimicrobial resistance was detected. Stronger biofilms were produced by MDR strains which is an important virulence determinant as well as a barrier against the treatment of infections. Biofilm formation of *S. aureus* isolates may be also associated with clonal linages associated with STs, *spa*- and *agr*-types since the differences among groups were consistent and statistically significant. The use of amikacin and tetracycline at concentrations corresponding to MIC or ten times higher were not sufficient to eradicate the biofilm which shows that biofilms constitute important barriers against the treatment of infections. Further studies will be carried out in order to investigate the presence and expression of biofilm-related genes and proteomic analysis of proteins associated with biofilm formation.

## Figures and Tables

**Table 1:** Biofilm-forming capacity and susceptibility to antimicrobials of the 23 selected strains.

Isolate	% of Biofilm Formation	Phenotypic Resistance/Susceptibility
D1	152.34%	PEN, KAN
D2	104.72%	KAN, TET
Dg1	122.09%	PEN
Dg2	95.86%	ERY
C1	138.52%	CIP
C2	108.54%	Susceptible
Rb1	197.61%	PEN, FOX, ERY, CD, CIP
Rb2	93.10%	PEN, FOX, ERY, CD, CIP, FD
Rt1	179.31%	PEN, FOX, ERY, CIP
Rt2	86.85%	Susceptible
Pi1	141.86%	PEN, CN, TOB, KAN, TET, C, CIP
Pi2	87.51%	PEN, FOX, ERY, CD, KAN, TET, C, CIP
B1	118.64%	Susceptible
B2	87.18%	FD
H1	147.98%	PEN, FOX, ERY, CD, CN
H2	114.81%	PEN, FOX, ERY, CD
Po1	162.72%	PEN, FOX, CN, TOB, KAN, ERY, CD, TET, CIP
Po2	109.58%	PEN, FOX, CIP, ERY, CD, TET, FD
O1	209.05%	Susceptible
O2	107.51%	Susceptible
Rt3 *mec*C	127.90%	PEN, FOX
Rt4 *mec*C	155.22%	PEN, FOX
O3 *mec*C	110.02%	PEN, FOX

D: donkey; Dg: dog; C: camel; Rb: rabbit; Rt: rodent; Pi: pig; B: bovine; H: hare; Po: poultry; O owl; PEN: penicillin; KAN: kanamycin; TET: tetracycline; ERY: erythromycin; CIP: ciprofloxacin; FOX: cefoxitin; CD: clindamycin; FD: fusidic acid; CN: gentamycin; TOB: tobramycin; C: chloramphenicol.

**Table 2:** Mean (M), standard deviation (SD) and univariate effects of biofilm formation by resistant to each antimicrobial.

Antimicrobial	Resistant M ± SD	Susceptible M ± SD	*p*
Penicillin	119.878 ± 22.831	116.624 ± 23.576	0.310
Cefoxitin	122.015 ± 24.605	115.7175 ± 20.661	0.023
Ciprofloxacin	126.632 ± 23.554	113.707 ± 19.932	<0.001
Gentamicin	123.539 ± 23.002	116.666 ± 22.044	0.036
Tobramycin	122.309 ± 21.526	117.651 ± 23.469	0.262
Kanamycin	123.997 ± 22.816	116.4612 ± 22.03	0.022
Erythromycin	124.634 ± 25.630	114.832 ± 19.881	0.001
Clindamycin	124.634 ± 25.630	115.536 ± 21.380	0.004
Tetracycline	119.381 ± 20.812	118.031 ± 24.293	0.689
Chloramphenicol	109.437 ± 15.221	119.551 ± 23.763	0.027
Fusidic acid	121.917 ± 23.568	118.219 ± 23.164	0.552

## Data Availability

Not applicable.
